# WNT-mediated gene gating: a novel principle connecting oncogenic super-enhancers with the nuclear pore to drive pathological expression of MYC

**DOI:** 10.1080/23723556.2019.1710992

**Published:** 2020-01-27

**Authors:** Anita Göndör

**Affiliations:** Department of Oncology-Pathology, Karolinska Institutet, Karolinska University Hospital, Bioclinicum, Solna, Sweden

**Keywords:** WNT signalling, Chromatin, nuclear architecture, nuclear pore, MYC super-enhancer

## Abstract

WNT signaling enhances *MYC* expression in cancer cells to increase the rate of cell proliferation. We have recently found that this principle involves the gating of *MYC* to nuclear pores mediated by an oncogenic super-enhancer in a ß-catenin-dependent manner in colon cancer cells. This phenomenon, which is absent in normal cells, leads to pathological levels of *MYC* expression.

Phenotypic plasticity is an important hallmark of cancer cells, as it underlies the ability of the tumor to evolve under changing selection pressure.^1^ This can occur at many levels, ranging from overstimulated cell signaling pathways to rewired chromatin states that synergistically abrogate normal controls of cell proliferation/differentiation.^2^ Although it is also well known that the nuclear architecture undergoes changes during the neoplastic process – a feature that is still used in cancer diagnosis – it is still an unchartered territory as to how all these features converge to create the cancer cell. An emerging point in case is an overactive WNT signaling, which has frequently been implicated in cancer evolution.^3^ One of the targets in this process is the *MYC* gene, which, when abnormally active, enhances cell proliferation by accelerating cell cycle progression. This feature has frequently been linked to the emergence of oncogenic super-enhancers (OSE), which can encompass several hundred kbs, upon which several different signaling pathways, such as that of WNT, converge to ensure *MYC* over-expression.^4^

Although such super-enhancers are generally considered to manifest transcriptional activation, this perspective might not fully encompass their mechanism of action. Another level of gene regulation involves a tantalizing, but poorly understood principle. The gene gating concept, first proposed >30 years ago, posits that the nuclear pore complexes act as organelles capable of interacting with a specific set of active genes to coordinate transcription, mRNA processing and nuclear export.^5^ As inducible genes are overrepresented among gated genes in yeast, it is generally considered that the gating principle functions to rapidly increase the expression levels of a subset of such genes in response to environmental cues.^6^ Although this principle has been documented in yeast, Drosophila and *C. elegans*, there is scant evidence for its existence in mammals, likely reflecting fundamental differences in higher order regulation of gene expression. For example, yeast transcription does not rely on long-range enhancers, and the volume of the yeast nucleus is >100-fold smaller than the mammalian counterpart to complicate direct comparisons between mammals and yeast. Nonetheless, indications that this principle operates also in mammalian cells were provided by the observations that NUP98 and NUP153, which form part of the nuclear pore, bind to a subset of super-enhancers that localize to the nuclear periphery.^7^

This reasoning was reinforced by our initial observation that a subpopulation of the alleles of the oncogenic colorectal super-enhancer (OSE), which is located more than 500 kb upstream of *MYC*, was juxtaposed to the nuclear pores in colon cancer cells.^8^ Using a combination of conventional and novel techniques, such as the Chromatin *in situ* Proximity (ChrISP) method that translates proximity between two DNA FISH probes or a DNA FISH probe and specific protein epitopes into fluorescent signal, it could thus be documented that the OSE is in close proximity to NUP133 epitopes at the nuclear periphery. Moreover, the potential for the interaction between the OSE and the transcriptionally active *MYC* gene was highest at the nuclear periphery/pore (Figure 1a).^8^ These and other observations pointed to a role for the OSE in mediating the juxtaposition of *MYC* and its transcripts to the nuclear pores. Although we could show that this process facilitated the nuclear export of *MYC* mRNA into the cytoplasm of colon cancer cells, but not in normal cell counterparts, it did not explain why colon cancer cells displayed several-fold higher levels of *MYC* mRNA than primary cultures of human colon epithelial cells. A compounding observation was that the actual transcription rate of *MYC* was paradoxically lower in the cancer cells than in normal cells to question the importance of the oncogenic super-enhancer in *MYC* transcription. The key observation resolving this enigma was our observation that *MYC* mRNAs decayed several-fold more rapidly in the nucleus than in the cytoplasm (Figure 1b),^8^ implying that the gating principle provides a rheostat to post-transcriptionally regulate cytoplasmic levels of *MYC* mRNA in response to environmental cues. Indeed, we could also show that the canonical WNT signaling pathway regulated the recruitment of the key component of a pre-nucleoporin complex, AHCTF1, to the OSE in a ß-catenin-dependent manner.^8^

**Figure 1.**
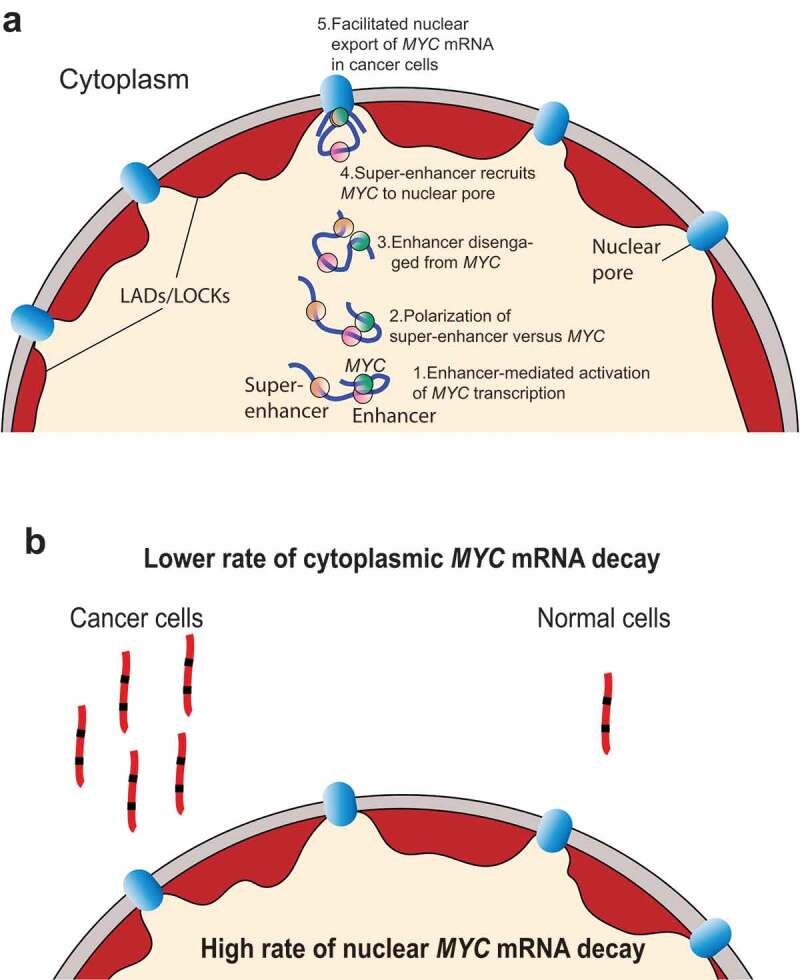
An oncogenic super-enhancer mediates the gating of *MYC* to the nuclear pore. (a) The scheme depicts that the colorectal, oncogenic super-enhancer dynamically migrates to the nuclear pore with the transcriptionally active *MYC* trailing behind (steps 1–3). Another enhancer more proximal to *MYC* and which does not bind to the nuclear pore is hypothesized to transcriptionally activate *MYC* in the nucleoplasm (steps 2–3). Prior to anchoring to the nuclear pore, which is specific for cancer cells, the potential for interactions between the oncogenic super-enhancer and *MYC* increases (step 4) to facilitate the nuclear export of *MYC* mRNA (step 5). While the scheme does not rule out a role for the oncogenic super-enhancer in transcriptional activation, the scheme highlights its post-transcriptional role. (b) Due to the several-fold higher rate of *MYC* mRNA decay in the nucleus, the facilitated export of nuclear *MYC* mRNA leads to pathological levels of *MYC* expression in the cytoplasm of specifically cancer cells.

These observations are generally in concordance with the gene gating principles observed in yeast and flies, although there are a few significant differences. Above all, the process is in human cancer cells likely very dynamic, reflecting the much larger volume of the human cell nucleus in comparison with the yeast and fly counterparts. The involvement of the OSE in mediating the gating principle is also unprecedented to raise a number of questions. For example, how is the WNT signaling pathway able to load nucleoporins to the OSE in cancer cells and not in the normal cell counterparts? What mechanism recruits the OSE to the nuclear periphery/pore and how is this regulated? One cue might be provided by the observation that the regions undergoing gating either contain or are flanked by so-called lamina-associated domains that are enriched in the repressive H3K9me2 mark. This thinking is reinforced by our earlier findings showing that inhibiting the enzyme G9a/Glp that lays down the H3K9me2 marks antagonized the migration of circadian genes to the nuclear periphery.^9^ Beyond *MYC*, it will be important to establish how general this phenomenon is and to what extent this principle is hijacked during cancer evolution. For example, there are reasons to suspect that *IgH* expression is also regulated by the gating principle to effectuate high levels of *IgH* expression in B cells. If so, the frequent translocation of *MYC* into the *IgH* enhancer region in B cell lymphomas might not only reflect transcriptional activation, but also its potential gating function. Given the enrichment of nucleoporin binding sites in super-enhancers,^7^ it will be interesting to explore whether the gating function is a general feature of these regulatory elements and how it is integrated with their function in transcriptional activation.

Taken together, our observations show that cancer cells can exploit the gene gating principle to increase the expression of an oncogene post-transcriptionally. This provides an opening to target *MYC* expression specifically in cancer cells without affecting *MYC* expression in normal cells of the patient. To achieve this, we need to document that this principle operates beyond *in vitro* cell model systems and to what extent it contributes to the adaptive phenotype of the evolving cancer cell.
